# Vascular Expression of Transient Receptor Potential Vanilloid 1 (TRPV1)

**DOI:** 10.1369/0022155415581014

**Published:** 2015-03-25

**Authors:** Claire A. Sand, Andrew D. Grant, Manasi Nandi

**Affiliations:** British Heart Foundation Centre for Cardiovascular Research, King’s College London, London, United Kingdom (CAS, MN); William Harvey Research Institute, Queen Mary University of London, London, United Kingdom (CAS); Wolfson Centre for Age-Related Diseases, King’s College London, London, United Kingdom (ADG); Pharmacology and Therapeutics, Institute of Pharmaceutical Science, King’s College London, London, United Kingdom (MN)

Dear Editor,

In the February 2014 issue of the Journal of Histochemistry & Cytochemistry, Tόth and colleagues published a report on Transient Receptor Potential Vanilloid 1 (TRPV1) expression and function in the rat vascular system (Tόth et al. 2014). The authors found that several commercially available anti-TRPV1 antibodies were not selective for TRPV1, and identified two that were considered to be individually selective for either neuronal or vascular—specifically smooth muscle—TRPV1. We have carried out similar studies on the role of TRPV1 in the mouse vascular system (which shares 95% nucleotide sequence homology with the rat), focusing specifically on endothelial cells. Like Tόth and colleagues, we found several commercially available anti-TRPV1 antibodies lacked specificity for TRPV1, highlighting the importance of conducting functional analysis of TRPV1 expression and activity. We found no evidence of functional TRPV1 expression in isolated murine endothelial cells and smooth muscle cells, despite numerous previous reports to the contrary. These data call into question much of what has been published on TRPV1 expression in the vasculature.

The role—and indeed the very presence—of TRPV1 in vascular tissue remain highly contentious. While several groups have reported evidence of endothelial TRPV1 expression (Bratz et al. 2008; Fantozzi et al. 2003; Yang et al. 2010), others have failed to reproduce these findings (Cavanaugh et al. 2011; Marrelli et al. 2007). Many studies have relied on mRNA expression alone, and protein quantification has often been conducted in the absence of appropriate controls, or using antibodies that have not been validated in TRPV1 knockout (KO) tissue. We aimed to clarify these discrepancies using a combination of biochemical and functional analysis in a number of different endothelial cell lines.

**Figure 1.**
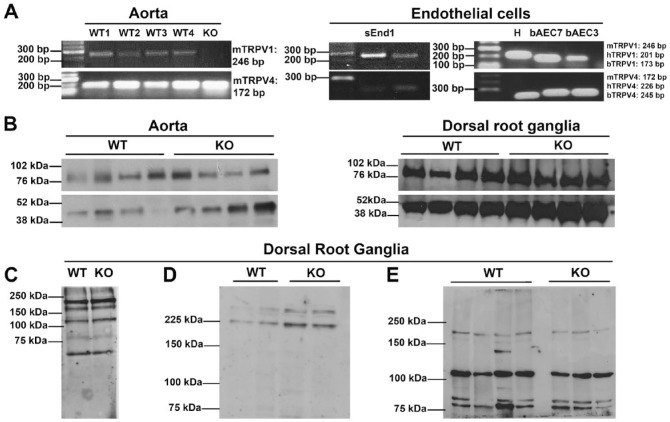
(A) Murine [m], human [h] and bovine [b] TRPV1 and TRPV4 mRNA in aortic lysates from four TRPV1 wild-type (WT1–4) mice and one TRPV1 knock out (KO) mouse (left panel), and in mouse skin endothelioma cells (sEnd1), human umbilical vein endothelial cells (H), and bovine aortic endothelial cells at passage 7 (bAEC7) and passage 3 (bAEC 3) (right panel). (B) Representative immunoblots of TRPV1 protein expression in murine aortic and dorsal root ganglia lysates from TRPV1 WT and KO mice, probed with ACC-030 anti-TRPV1 antibody (Alomone Labs, Jerusalem, Israel; predicted molecular weight, 95 kDa). β-actin expression was used as a loading control (predicted molecular weight, 42 kDa). (C–E) Representative immunoblots of dorsal root ganglia lysates from TRPV1 WT and KO mice using a number of different anti-TRPV1 antibodies: (C) ab4579, Abcam (Cambridge, UK); (D) ACC-030 Batch 2, Alomone Labs; (E) V2764, Sigma-Aldrich (St Louis, MO).

Using reverse transcription and PCR amplification of vascular cDNA, we found evidence— consistent with the observations of Tόth and colleagues—of TRPV1 mRNA expression in aortic lysates of wild type (WT), but not TRPV1 KO, mice and in freshly isolated and immortalized endothelial cells from three different species (Fig. 1A). TRPV4 expression, here used as a positive control, was clearly evident in all tissues (Fig. 1A), consistent with published reports (Baylie and Brayden 2011). In order to confirm protein expression, we used ACC-030 (Alomone Labs; Jerusalem, Israel), a widely used anti-TRPV1 antibody that, in previous reports, showed no immunoreactivity in samples from TRPV1 KO mice (Yang et al. 2010); albeit, this was in the absence of a protein loading control. We observed clear and distinct bands in aortic and dorsal root ganglia lysates of KO mice of identical origins to those used by Yang et al. (Fig. 1B), suggesting— in line with the results of Tόth and colleagues—that this antibody is not a suitable indicator of TRPV1 protein expression. Furthermore, these bands were observed at approximately 75 kDa, which is 20 kDa smaller than the predicted molecular weight of TRPV1 (Caterina et al. 1997); this is further evidence of non-selective immunoreactivity. We additionally tested three other commercially available antibodies (Table 1) that were similarly non-selective for TRPV1 (Fig. 1C–1E). Although TRPV1 KO mice are functional knockouts and retain the C-terminus of the TRPV1 gene against which most TRPV1 antibodies are raised, sequencing of TRPV1 KO cDNA revealed a TAG stop codon within the open reading frame of the inserted neomycin cassette, precluding the possibility of residual C-terminal translation. These observations call into question much of the data on TRPV1 expression that has been published to date, and support the observations of Tόth and colleagues that numerous commercially available antibodies are not selective for TRPV1.

**Table 1.** Antibodies Used in Western Blot Analysis.

Specificity	Region	Manufacturer	Catalog No	Species	Vehicle	Working Dilution
*Primary Antibodies*						
TRPV1	C-terminal	Alomone Labs (Jerusalem, Israel)	ACC-030	Rabbit	1% BSA, 0.05% NaN_3_ in PBS	1:200
TRPV1	C-terminal	Abcam (Cambridge, UK)	Ab45759	Mouse	1% BSA, 0.05% NaN_3_ in PBS	1:200
TRPV1	C-terminal	Sigma-Aldrich (St Louis, MO)	V2764	Rabbit	1% BSA, 0.05% NaN_3_ in PBS	1:500
β-actin	N-terminal	Sigma-Aldrich	A2228	Mouse	1% non-fat milk in TBS-T	1:2000
*Secondary Antibodies*						
Rabbit IgG		Cell Signaling Technology (Beverly, MA)	7074		1% non-fat milk in TBS-T	1:2000
Mouse IgG		Sigma-Aldrich	A4416		1% non-fat milk in TBS-T	1:5000

BSA, bovine serum albumin; PBS, phosphate-buffered saline; TBST, Tris-buffered saline containing 1% Tween-20.

**Figure 2.**
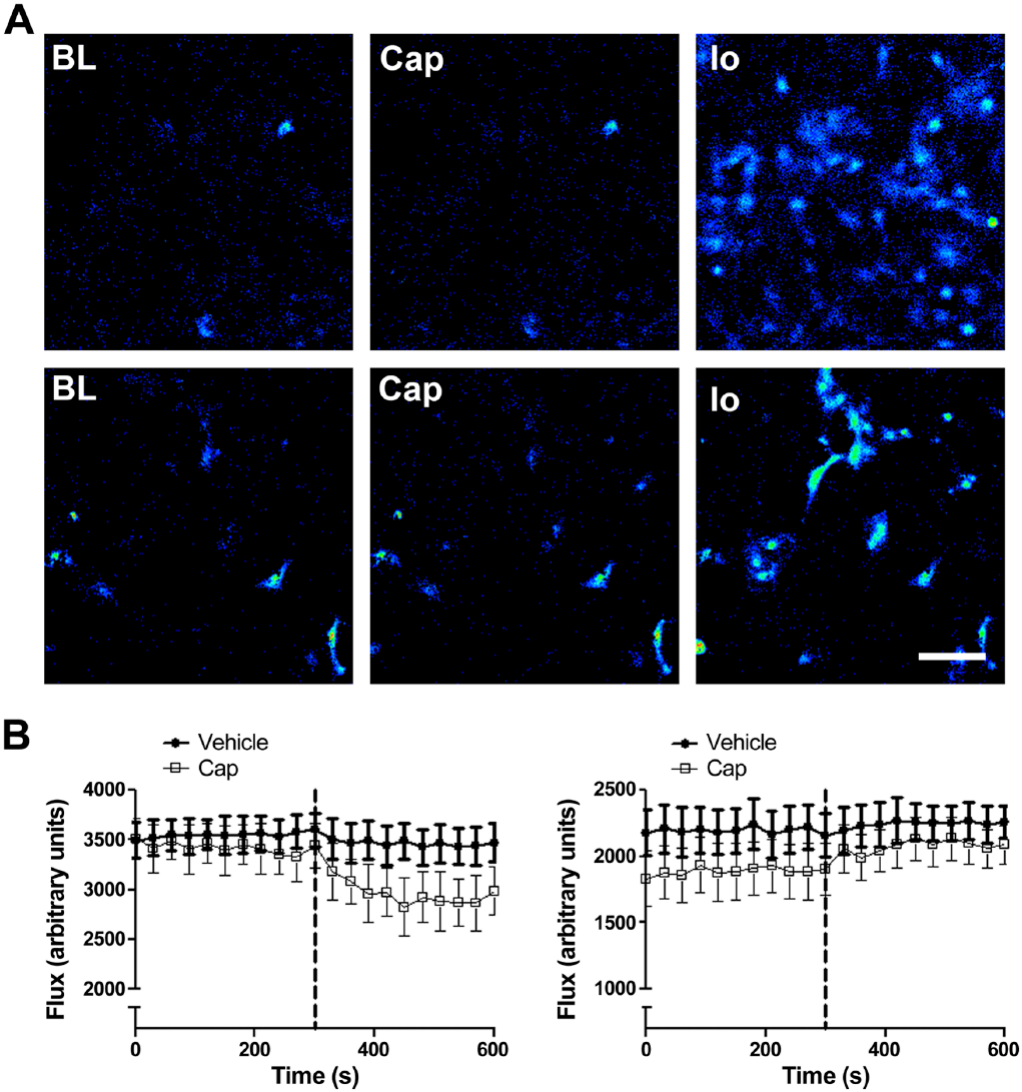
(A) Capsaicin-induced calcium fluorescence in murine pulmonary endothelial cells (upper panel) and murine aortic smooth muscle cells (lower panel). Representative images were captured at baseline (BL), and after stimulation with 1 µM capsaicin (Cap), and 1 µM ionomycin (Io). No increase in intracellular Ca^2+^ was observed in either endothelial or smooth muscle cells in response to 1 µM capsaicin. Scale, 40 µM. (B–C) Blood flow responses in first-order mesenteric vessels treated with capsaicin and vehicle (2% DMSO in saline) in healthy and endotoxaemic (LPS; 12.5 mg/kg, i.v., 24 hr) wild type (WT) mice, respectively. Baseline mesenteric blood flow was recorded for 5 min; capsaicin (Cap; 10 µM) or vehicle (2% DMSO in saline) was then administered as an aerosolized spray, denoted by the dotted line, and blood flow was recorded for a further 5 min. In naïve mice, capsaicin caused a decrease in blood flow, indicative of vasoconstriction; in LPS-treated mice, however, capsaicin increased blood flow. Data are presented as mean ± SEM (*n*=6–14).

In the absence of a biochemical method for determining TRPV1 protein levels, we attempted to assess TRPV1 expression and activity by functional assay using the highly selective agonist capsaicin. Freshly isolated murine pulmonary endothelial cells and aortic vascular smooth muscle cells were perfused with 1 µM capsaicin, roughly equivalent to the EC_50_ of rat TRPV1 in heterologous expression systems (Caterina et al. 1997; Welch et al. 2000). Neither cell type exhibited any calcium influx in response to capsaicin treatment, despite responding robustly to the positive control, ionomycin (Fig. 2A). Our functional observations are consistent with the lack of vasoactive responses to capsaicin in rat aortae reported by Tόth and colleagues (Tόth et al. 2014). Therefore, despite detectable mRNA expression in murine aorta, immortalized murine endothelial cells and bovine aortic endothelial cells (Fig. 1A), no evidence of functional TRPV1 expression was observed during the functional analysis. This was an unexpected observation, given previous reports of significant Ca^2+^ influx in murine aortic endothelial cells in response to 1 µM capsaicin (Yang et al. 2010). It is possible that TRPV1 expression is tightly regulated at the level of the blood vessel, as suggested by Tόth and colleagues, and that the cells studied were not appropriate for the detection of vascular TRPV1 activity.

It is similarly possible, given that TRPV1 KO mice appear to have a normal hemodynamic profile under basal conditions (Marshall et al. 2013; Pacher et al. 2004), that protein translation and activity in some vascular tissue are minimal in the physiological setting, and that, under pathological conditions, the receptor is upregulated, as observed previously in other cell types (Ständer et al. 2004). A scenario in which endothelial cells retain a pool of TRPV1 mRNA that is translated under conditions of stress or inflammation may explain our observations of clear TRPV1 mRNA expression despite no evidence of receptor activity.

In contrast with the results of Tόth and colleagues, but consistent with previous findings (Scotland et al. 2004), we observed a decrease in mesenteric blood flow (indicative of vasoconstriction) in response to the topical administration of capsaicin (10 µM in 2% DMSO, delivered by aerosolized spray) in a previously described (Sand et al. 2014) in situ model of microvascular perfusion monitoring (Fig. 2B). This method, conducted under a UK Home Office licence, following local ethics committee approval, and in accordance with the Home Office Animal (Scientific Procedures) Act, 1986, allows in situ real-time laser speckle contrast imaging of mesenteric vasoactive responses to pharmacological stimuli, without the need for microvascular dissection and organ bath preparation. A topical method of application was chosen to assess vasoactive responses in mesenteric vessels directly. A relatively high concentration of capsaicin [10 µM – approximately 10-fold higher than the EC_50_ in heterologous expression systems (Caterina et al. 1997)] was chosen for topical administration, owing to the relatively diffuse delivery method. The reasons for the divergent observations are unclear. Whereas Tόth and colleagues did report immunoreactivity with the anti-TRPV1-N antibody in the vascular smooth muscle layer of rat mesenteric arteries, the authors did not observe any functional responsiveness to capsaicin, and, somewhat confusingly, did not detect any TRPV1 mRNA in mesenteric arteries.

Whether our observations of decreased mesenteric blood flow in response to capsaicin application are due to activation of smooth muscle receptors or to the release of constrictor neuropeptides from sensory nerve terminals is not clear. Although activation of neuronal TRPV1 is generally associated with the release of calcitonin gene-related peptide (CGRP), a highly potent vasodilator (Brain et al. 1986), it is possible, as suggested previously (Scotland et al. 2004), that capsaicin-induced mesenteric constriction is mediated by an alternative constrictor neuropeptide. It is also possible that certain mediators only present in situ (and absent in organ bath preparations) are responsible for facilitating TRPV1 activation in mesenteric vascular smooth muscle cells. Interestingly, in endotoxemic mice treated systemically with lipopolysaccharide (LPS; 12.5 mg/kg, i.v. 24 hr previously), the vasoconstrictor response to capsaicin was abolished, and we observed a subtle vasodilatation (Fig. 2C), suggesting that the expression profile of either TRPV1 or its downstream mediators may be altered in inflammation. It is possible, therefore, that inflammation-induced upregulation of vasodilator peptides, such as CGRP (known to be elevated in sepsis), may be sufficient to overcome any constriction mediated by the activation of smooth muscle TRPV1.

Despite demonstrating robust TRPV1 mRNA expression in aortic lysates and isolated endothelial cells, we have found no evidence of functional TRPV1 protein expression in these tissues; this finding contrasts with numerous previous reports to the contrary. On the other hand, these data do not preclude the possibility of induced TRPV1 expression under certain pathological conditions, the presence of vanilloid-insensitive TRPV1 splice variants, or, indeed, the presence of functional expression in specific subpopulations of vascular cells, as demonstrated by Tόth and colleagues. Our findings in the human, cow and mouse corroborate and add to the results obtained in rat tissue by Tόth and colleagues. Our use of TRPV1 KO tissue to assess antibody specificity also represents a more robust negative control than blocking antibodies. We additionally extended this work into the functional analysis of TRPV1 expression. Although our in vivo results were potentially consistent with the smooth muscle expression of TRPV1 reported by Tόth and colleagues, we were unable to confirm this in isolated cells (perhaps due to artefacts of culture and isolation).
